# Circulating miRNA‐375 as a potential novel biomarker for active Kaposi’s sarcoma in AIDS patients

**DOI:** 10.1111/jcmm.14054

**Published:** 2018-12-13

**Authors:** Maria Assunta Piano, Lisa Gianesello, Angela Grassi, Paola Del Bianco, Adriana Mattiolo, Anna Maria Cattelan, Lolita Sasset, Paola Zanovello, Maria Luisa Calabrò

**Affiliations:** ^1^ Immunology and Molecular Oncology Veneto Institute of Oncology IOV ‐ IRCCS Padova Italy; ^2^ Clinical Trials and Biostatistics Veneto Institute of Oncology IOV ‐ IRCCS Padova Italy; ^3^ Infectious and Tropical Diseases Azienda Ospedaliera and University of Padova Padova Italy; ^4^ Infectious Diseases ULSS 18 ‐ Azienda Ospedaliera Rovigo Italy; ^5^ Department of Surgery, Oncology and Gastroenterology University of Padova Padova Italy; ^6^ Present address: Clinical Nephrology, Department of Medicine University of Padova Padova Italy

**Keywords:** biomarker, circulating miRNA, HHV8, HIV, Kaposi’s sarcoma, microRNA, miR‐375

## Abstract

The aim of this study was to identify circulating microRNAs (miRNAs) that could be used as biomarkers in patients at risk for or affected by AIDS‐Kaposi's sarcoma (KS). Screening of 377 miRNAs was performed using low‐density arrays in pooled plasma samples of 10 HIV/human herpesvirus 8 (HHV8)‐infected asymptomatic and 10 AIDS‐KS patients before and after successful combined antiretroviral therapy (cART). MiR‐375 was identified as a potential marker of active KS, being the most down‐regulated in AIDS‐KS patients after cART and the most up‐regulated in naïve AIDS‐KS patients compared to naïve asymptomatic subjects. Validation on individual plasma samples confirmed that miR‐375 levels were higher in AIDS‐KS compared to asymptomatic patients, decreased after cART‐induced remission in most AIDS‐KS patients and increased in patients with active KS. In asymptomatic patients miR‐375 was up‐regulated after cART in both screening and validation. Statistical analyses revealed an association between miR‐375 changes and CD4 cell counts, which could explain the discordant cases and the opposite trend between asymptomatic and AIDS‐KS patients. These data suggest that circulating miR‐375 might be a good indicator of active AIDS‐KS. Moreover, changes in miR‐375 levels may have a prognostic value in HIV/HHV8‐infected patients undergoing treatment. Further large‐scale validation is needed.

## 1 introduction

Body fluids, such as plasma, were shown to harbour highly stable, either exosome‐associated or vesicle‐free, microRNAs (miRNAs), small noncoding regulatory RNA molecules crucially involved in several physiological and pathological cellular processes.^1^, ^2^ This finding opened up new venues for cancer diagnosis and prognosis, and circulating miRNA profiles or specific miRNAs have been proposed as informative and noninvasive biomarkers for several tumours, though it remains to be clarified whether they originate from tumour cells or from nonmalignant cell types as a response to the pathological status.^3^

Kaposi's sarcoma (KS) is a multifocal angioproliferative soft tissue sarcoma, involving the skin and mucosa, associated with human herpesvirus 8 (HHV8) infection. HHV8 is also linked to the development of primary effusion lymphoma (PEL), a non‐Hodgkin's B‐cell lymphoma.^4^ Immunocompromised HIV/HHV8‐infected patients are at high risk for KS. Indeed, although the incidence of AIDS‐defining cancers has declined consistently in the era of antiretroviral therapy (ART), KS remains 650 times more common in HIV‐infected persons compared to uninfected subjects.^5^

Initial studies on PEL‐derived cell lines, KS biopsies and HHV8‐infected or HHV8‐transformed endothelial cells led to the identification of virus‐modulated or tumour‐specific cell‐associated miRNAs.^6^ Subsequent analyses of KS biopsies and healthy tissues provided contrasting data, with some miRNAs showing an opposite trend.^6^, ^7^, ^8^ Exosomes isolated from PEL effusions, pooled plasma samples of AIDS‐KS patients and two KS mouse models were found to deliver HHV8‐encoded and host oncogenic miRNAs triggering cell migration and IL‐6 release,^9^ highlighting their function in the context of HHV8‐driven tumorigenesis. To date, studies aimed at identifying circulating miRNAs as blood‐based biomarkers for KS diagnosis and prognosis have not been carried out. To this end, we conducted a pilot study on plasma samples of patients at risk for or affected by AIDS‐KS.

## 2 materials and methods

### 2.1 Study population

Two groups of 10 HIV/HHV8‐seropositive patients were selected for the initial screening: HIV‐seropositive patients with serological and molecular evidence of HHV8 infection, and without clinical signs of HHV8‐related and ‐unrelated pathologies, asymptomatic patients (group A), and patients affected by AIDS‐KS (group B). Criteria for patient inclusion were: availability of never‐thawed plasma samples in sufficient amount at baseline (naïve) and after immunovirological response to combined ART (cART), histological confirmation of KS and clinical response (complete remission, CR) to cART for AIDS‐KS patients. Seventeen patients received cART consisting of two nucleoside reverse transcriptase inhibitors (NRTIs) in combination with a boosted protease inhibitor (PI), whereas three patients received two NRTIs in combination with a non‐nucleoside reverse transcriptase inhibitor (NNRTI). Validation included 11 asymptomatic individuals (three belonging to the screening phase and eight newly selected), and 11 AIDS‐KS patients (six newly selected). In addition, four AIDS‐KS patients with disease progression (DP) or partial response (PR) were selected (“non‐responder AIDS‐KS”), including one patient with paradoxical KS‐associated immune reconstitution inflammatory syndrome (KS‐IRIS) and one patient with unmasking KS‐IRIS. These patients received two NRTIs in combination with a boosted PI.

Blood samples were obtained after written informed consent from patients attending the Unit of Infectious Diseases at the General Hospitals of Padova and Rovigo. The samples were then analysed for HHV8 infection at the Veneto Institute of Oncology. Patients were asked to donate the residual part of the plasma sample for research purposes, as well as the residual part of previously collected samples. The proposed research project was evaluated and approved by the local Ethical Committee (n. 14256‐CE IOV 12/60).

### 2.2 Blood sampling and immunovirological assays

Plasma was obtained from EDTA‐treated peripheral blood samples after Ficoll‐Paque^TM^ PLUS (GE Health Care, Uppsala, Sweden) density gradient centrifugation. Plasma was recovered from the upper phase and centrifuged to ensure a cell‐free specimen, and subsequently aliquoted and stored at −80°C until use. For the screening analysis, plasma samples were pooled using 100 µl of plasma from each patient included in the four categories. Each pool was prepared, extracted, and analysed on a card, and this procedure was repeated twice from the beginning, to have a full technical duplicate for each group of pooled plasma. We could not perform a third replicate because of the limited amount of some plasma samples. In the validation phase, plasma samples were individually tested for the presence of the candidate and endogenous miRNAs.

Immunovirological parameters related to HIV infection, plasma HIV RNA, and CD4 cell counts, were assessed as previously reported^10^ and expressed as log_10_ HIV RNA copies/mL and CD4 cells/μL, respectively.

HHV8 antibodies to a latency‐associated nuclear antigen (LANA) were evaluated by indirect immunofluorescence, while lytic‐phase‐associated antibodies to the capsid‐related protein encoded by open reading frame (ORF)‐65 were tested by the enzyme‐linked immunosorbent assay (ELISA) as previously described.^11^ HHV8 antibody titres were determined using 1:50 as first dilution and expressed as reciprocal of the highest dilution giving a positive result. HHV8 load was determined in peripheral blood mononuclear cells (PBMC) and saliva samples by quantitative real time‐PCR and expressed in genome equivalents (GE).^12^

### 2.3 RNA extraction

miRNA extraction efficiency from plasma samples (pooled or not) was preliminarily assessed using four different methodologies: total RNA extraction with RNA Bee (Tel‐Test Inc, Friendswood, TX, USA) and mirVana PARIS Kit (Life Technologies, Thermo Fisher Scientific, Waltham, MA, USA), small RNA molecule extraction with mirVana PARIS Kit, and Nucleospin miRNA Plasma Kit (Macherey‐Nagel, GmbH & Co, Germany). RNA concentration and purity was assessed using Nanodrop 1000 (Thermo Scientific, Wilmington, DE, USA) and RNA integrity using a 2100 Bioanalyzer (Agilent Technologies, Santa Clara, CA, USA). RNAs extracted with the four techniques were then analysed using the TaqMan miRNA assays (Life Technologies, Thermo Fisher Scientific, Carlsbad, CA, USA) specific for U6 and RNU48. On the basis of RNA purity, integrity, and amplification plots, we selected the Nucleospin miRNA Plasma Kit as the most efficient method to extract total plasmatic miRNAs, including those incorporated into exosomes, according to the manufacturer's instructions. Total RNA, enriched in low molecular weight molecules, was therefore extracted using NucleoSpin miRNA columns from the pooled plasma belonging to each group. RNA concentration, purity, and integrity were evaluated as described above. Extracted RNA was then analysed for U6 using a TaqMan microRNA assay (Life Technologies) to further evaluate the quality and miRNA amplication of the extracted small‐sized RNA fraction.

### 2.4 Statistical analysis

Only measurable samples were considered for descriptive purposes whereas data under the detection level were replaced with a constant value (40 for HIV viraemia, 4.5 GE for HHV8 viral load, 45 for HHV8 antibody titres) for statistical analysis purposes.

All patient characteristics before and after therapy were summarized as median and interquartile range expressed as (Q1;Q3), and compared using the Kruskal‐Wallis test to assess whether the screening and validation datasets had the same median.

The differential distribution of all parameters at baseline, after therapy and their change from baseline, between AIDS‐KS and asymptomatic patients was assessed by the Kruskal‐Wallis test, and the pairwise comparisons between levels at baseline and after therapy within each group of patients was based on the Wilcoxon signed rank test.

The association between all parameters and RQ levels, dichotomized as higher and lower than 1, was verified with the Kruskal‐Wallis test. All statistical tests used a two‐sided 5% significance. Statistical analyses were performed using the sas statistical package (SAS, rel. 9.4; SAS Institute Inc).

### 2.5 miRNA profiling

Global miRNA profiling was performed by real‐time quantitative PCR using an ABI Prism 7900HT sequence detector system (Applied Biosystems, Thermo Fisher Scientific, Waltham, MA USA). Reverse transcription using 500 ng of total RNA was performed using the TaqMan MicroRNA Reverse Transcription Kit (Applied Biosystems) in combination with the Megaplex RT human Primers Pool A (Applied Biosystems) according to the manufacturer's protocol. cDNA was loaded onto 384 well TaqMan low‐density arrays (Human MicroRNA Array A, panel v2.0, Applied Biosystems) for the study of 377 well‐known and highly characterized miRNAs. Amplification results were analysed with SDS 2.4 and RQ Manager 1.2.1 (Life Technologies). The amplification curves were individually inspected and miRNAs with abnormal amplification patterns were removed from analysis. Undetermined Ct values were set to a maximum value of 40. All the miRNAs whose Ct standard deviation exceeded the 75‐th percentile of the observed standard deviation distribution were not considered in the differential expression analysis, in order to control the number of unreliable discoveries due to technical variability.

The three miRNAs expressed in all the four conditions and showing the lowest standard deviation of raw Ct values were selected as candidate endogenous references. Relative expression of target miRNAs was calculated as ΔCt_miRNA_ = Ct_miRNA_ – Ct_endogenous reference._ Differential expression of each miRNA was then expressed as ΔΔCt_miRNA_ = ΔCt_miRNA_^treated^ – ΔCt_miRNA_^naïve^, by subtracting from the ΔCt of a specific miRNA in group “treated” the ΔCt of that miRNA in group “naïve”. The fold change (FC) was calculated as 2^–ΔΔCt^ assuming a PCR amplification efficiency of 100%.^13^ Analyses of TaqMan low‐density array microRNA data were performed in the R software suite.

### 2.6 qRT‐PCR validation

Validation analysis for the selected miRNAs was performed by quantitative real‐time RT‐PCR in 11 individual plasma samples from each group. miRNA‐specific reverse transcription of 10 ng RNA for each sample was performed using the TaqMan MicroRNA Reverse Transcription Kit (Applied Biosystems) and cDNA were amplified using specific miRNA TaqMan assays (miR‐375 ID: 000564; miR‐92a ID: 000431) according to the manufacturer's instructions. The assays were performed in duplicate on the ABI 7900HT RealTime PCR system (Applied Biosystems). miR‐375 relative quantification (RQ) was done using miR‐92a as endogenous control with the 2^–∆∆Ct^ method^13^: 2^–ΔΔCt^ = 2^–[ΔCtmiRNA (treated) – ΔCtmiRNA (naïve)]^. Normalization based on the global mean was performed as previously described.^14^

## 3 RESULTS

### 3.1 Analysis of patient characteristics

Twenty HIV/HHV8‐infected patients were selected for the initial screening and included 10 asymptomatic patients (group A), naïve and cART‐treated, and 10 AIDS‐KS subjects (group B), naïve and cART‐treated; 22 HIV/HHV8‐infected patients were selected for the validation phase and included 11 asymptomatic subjects (group A), naïve and cART‐treated, and 11 AIDS‐KS subjects (group B), naïve and cART‐treated. Demographics and immunovirological parameters of all study patients are reported in Table 1.

**Table 1:** Patient characteristics

Patients	Gender total (M/F)	Age Median (Q1;Q3)	HIV‐ and HHV8‐related parameters^a^						Treatment months (Q1;Q3)
			CD4 cell count (cells/µL)	HIV plasma load (log_10_ copies/mL)	HHV8 PBMC load (log_10_ GE/10^5^ cells)	HHV8 saliva load (log_10_ GE/10^5^ cells)	HHV8 ORF65 antibodies^b^	HHV8 LANA antibodies^b^	
Naïve asymptomatic (group A)									
Screening	10 (9M/1F)	39 (34;43)	270 (141;332)	4.86 (4.15;5.39)	^(5;5;0) ^1.52 (1.18;1.90)	^(5;5;0) ^2.26 (2.21;2.89)	^(4;6;0) ^50 (50;63)	^(9;1;0) ^200 (50;800)	
Validation^c^	11 (8M/3F)	36 (33;46)	362 (285;452)	5.04 (4.08;5.23)	^(6;5;0) ^1.44 (1.24;1.93)	^(9;2;0) ^2.26 (1.74;2.45)	^(5;6;0) ^50 (50;50)	^(10;1;0) ^50 (50;1250)	
*P*‐value		0.8321	0.0669	0.7511	0.8229	0.2978	0.9678	0.6328	
cART‐treated asymptomatic									
Screening	10 (9M/1F)	40.5 (35;44)	650 (408;687)	^(3;7;0) ^1.78 (1.75;1.97)	^(1;9;0) ^0.85	^(4;6;0) ^3.97 (3.04;4.52)	^(4;6;0) ^150 (88;250)	^(9;1;0) ^200 (50;1600)	20.5 (12;25)
Validation	11 (8M/3F)	37 (35;47)	671 (507;721)	<1.7	^(2;9;0) ^1.41 (1.05;1.77)	^(4;7;0) ^3.62 (2.32;4.77)	^(3;8;0) ^100 (75;150)	^(10;1;0) ^150 (50;1300)	19 (14;28)
*P*‐value		0.7768	0.5490	0.0564	0.6028	0.8403	0.4752	0.9422	0.5254
Naïve AIDS‐KS (group B)									
Screening	10M	41.5 (30;53)	210 (90;370)	5.2 (4.97;5.63)	^(4;4;2) ^2.21 (2.00;2.50)	^(5;3;2) ^4.12 (1.85;4.15)	^(9;1;0) ^400 (100;800)	^(9;1;0) ^1600 (400;3200)	
Validation^c^	11M	38 (30;47)	230 (90;381)	5.16 (4.97;6)	^(6;4;1) ^1.14 (1.04;2.77)	^(3;1;7) ^1.85 (1.62;3.13)	^(6;0;5) ^400 (175;700)	^(6;0;5) ^2400 (700;10 400)	
*P*‐value		0.8599	0.9718	0.9437	1	0.7946	0.6944	0.3486	
cART‐treated AIDS‐KS									
Screening	10M	42 (31;53)	343.5 (194;432)	^(3;7;0) ^2.11 (1.93;2.18)	^(4;4;2) ^1.26 (1.08;1.48)	^(7;1;2) ^4.30 (3.45;4.83)	1200 (100;6400)	^(9;0;1) ^3200 (1600;6400)	10 (7;13)
Validation	11M	39 (31;48)	390 (181;620)	^(3;8;0)^1.81 (1.78;2.68)	^(5;4;2) ^1.85 (1.18;1.90)	^(4;1;6) ^3.79 (3.11;4.31)	^(5;0;6) ^1600 (800;3200)	^(5;0;6) ^1600 (1600;6400)	9 (6;14)
*P*‐value		0.8597	0.6466	0.8946	0.6110	0.4175	0.7571	0.7841	0.9717
Non‐responder AIDS‐KS									
Naïve	4M	36 (30.25;43)	365 (229;555)	4.75 (4.2;5.5)	^(3;0;1) ^1.88 (1.57;1.94)	^(2;0;2) ^3.38 (2.6;4.2)	^(3;0;1) ^800 (425;1200)	^(3;0;1) ^12 800 (8000;19 200)	
cART‐treated	4M	40.5 (37.8;43.2)	^(2;0;2) ^777 (770;783)	^(1;1;2) ^2.05	^(1;3;0) ^0.85	^(3;1;0) ^4.12 (2.6;4.4)	1600 (1212;7600)	^(3;1;0) ^3200 (2000;8000)	

KS, Kaposi's sarcoma; Q1, first quartile; Q3, third quartile; HIV, Human immunodeficiency virus; HHV8, Human herpesvirus 8; PBMC, peripheral blood mononuclear cells; GE, genome equivalents; ORF65, open reading frame 65, encoding for a structural protein expressed during the lytic phase; LANA, latency‐associated nuclear antigen; cART, combined antiretroviral therapy.

a Medians and quartiles were calculated on the measurable samples. The three numbers between parentheses refer to samples with: measurable values; values under detection level; data not available.

b Antibody titres were determined using 1:50 as first dilution, and are expressed as reciprocal of the highest dilution giving a positive result.

c In the validation phase, asymptomatic patients included six newly selected patients and five individuals of the screening analysis, whereas AIDS‐KS subjects included eight newly selected individuals. All patient characteristics were compared using the Kruskal‐Wallis test.

No statistically significant differences in all variables, at baseline and after therapy, were found between screening and validation datasets (Table 1).

All variables were then compared between asymptomatic and AIDS‐KS patients, at baseline and after treatment (Table S1). CD4 cell counts were found to be significantly higher in cART‐treated asymptomatic subjects compared to cART‐treated AIDS‐KS patients in both the screening (median [Q1;Q3], 650 [408;687] vs 343.5 [194;432], *P* = 0.0233) and validation (671 [507;721] vs 390 [181;620], *P* = 0.0385) datasets, whereas HIV RNA loads did not significantly differ in each comparison. Concerning HHV8‐related parameters, a higher HHV8 load was observed in treated AIDS‐KS patients compared to treated asymptomatic patients in the screening set, but the difference did not reach significance. HHV8 antibody titres were significantly higher before and after treatment in AIDS‐KS patients compared to asymptomatic patients of the screening and validation groups, with the exception of LANA antibody levels between naïve AIDS‐KS and asymptomatic patients of the screening group (Table S1). Treatment duration was longer in asymptomatic compared to AIDS‐KS patients, but the difference was significant only in the validation group (screening: median months [Q1;Q3], 20.5 [12;25] vs 10 [7;13], *P* = 0.0628; validation: 19 [14;28] vs 9 [6;14], *P* = 0.0103).

Analysis of the changes after treatment (Table S1) indicated that antibody titres against ORF65 significantly increased in AIDS‐KS patients compared to asymptomatic patients in both screening (*P* = 0.0305) and validation (*P* = 0.0076) groups. Conversely, the increase in CD4 cell counts was significantly higher in asymptomatic patients compared to AIDS‐KS patients after treatment only in the screening (344.5 [174;386] vs 113.5 [80;160] *P* = 0.0025).

On the whole, these analyses indicated that screening and validation datasets did not differ for the considered variables, and that CD4 cell counts and HHV8 antibody titres were significantly different between treated asymptomatic and AIDS‐KS patients in screening and validation datasets.

### 3.2 Screening by low‐density arrays and identification of a candidate miRNA

Selected patients had a successful cART response, with a statistically significant decrease in HIV viraemia and a significant increase in CD4 cell counts (see Figure S1). Moreover, ORF65 antibody titres significantly increased in AIDS‐KS patients after treatment (Wilcoxon signed rank test, *P* = 0.0156), whereas all other parameters did not statistically change (not shown).

TaqMan low‐density arrays were used to screen 377 miRNAs in plasma samples, which were pooled to look for substantial characteristics of each patient group and to reduce differences due to subject‐to‐subject variation.

To identify stably expressed miRNAs to be used as endogenous controls, Ct variability of each miRNA across the different groups of patients was evaluated. Among the detectable miRNAs with Ct < 30, miR‐92a, miR‐320 and miR‐484 were the three most stable and were selected as reference miRNAs to normalize the Ct values of the remaining miRNAs in the cards. Differentially expressed miRNAs found in each comparison were consistent, independently from the chosen reference miRNA.

**Figure 1:**
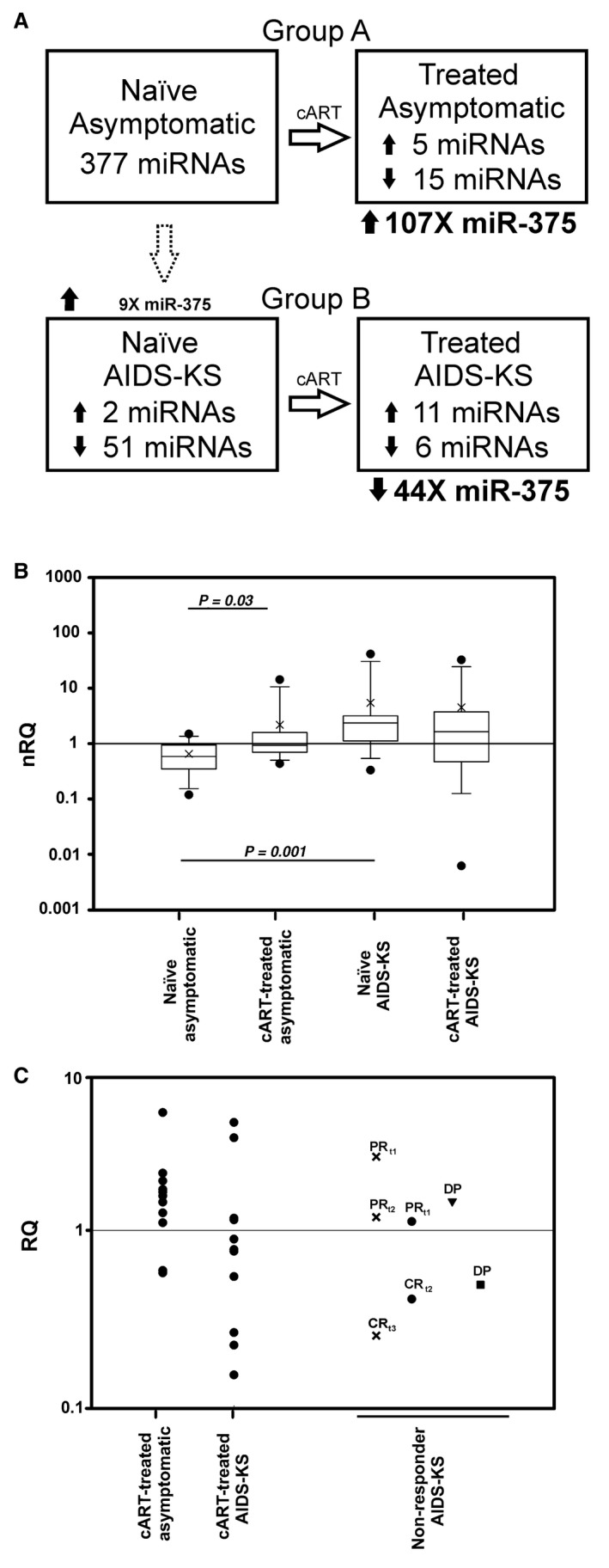
Modulated circulating miRNAs in the different patient groups of the screening analysis and validation of the candidate miRNA. A, The screening was conducted by analysing the expression levels of 377 miRNAs in HIV/HHV8‐infected patients, without and with Kaposi's sarcoma, before (naïve condition) and after successful antiretroviral treatment (cART‐treated). The number of modulated miRNAs found in each comparison of interest (naïve B vs naïve A, cART‐treated A vs naïve A, and cART‐treated B vs naïve B) are reported in each box. Circulating miR‐375, one of the most markedly modulated miRNA after cART, was chosen as candidate prognostic miRNA for subsequent validation. Reported fold changes of miR‐375 are calculated using miR‐92a as reference. B, Comparison of the levels of circulating miR‐375 in individual plasma of the four categories of patients. In the validation phase, naïve AIDS‐KS patients showed the highest median level of miR‐375 compared to the other three patient categories, suggesting that this miRNA might be indicative of active KS. Significant variations are indicated (Wilcoxon rank‐sum test). Cross symbols indicate the mean values. nRQ: normalized relative quantification. C, Variation of miR‐375 expression in patients analysed after successful cART and in AIDS‐KS patients not responding to therapy. In this graph, the expression level (RQ) is normalized with respect to the naïve condition: RQ > 1 is therefore indicative of increasing miR‐375 levels, whereas RQ < 1 indicates decreasing miRNA levels with respect to the baseline condition. MiR‐375 up‐regulation was found in most asymptomatic patients, whereas most AIDS‐KS patients showed miR‐375 down‐regulation, confirming the trend observed in the screening by low‐density arrays. On the right‐hand side of the Figure, 4 non‐responder AIDS‐KS patients were analysed in follow‐up (indicated with t for different time‐points after cART). Two AIDS‐KS patients (x symbol and solid circle) during partial response (PR) showed RQ > 1, and subsequently RQ < 1 following complete remission (CR). A patient with disease progression (DP) who developed paradoxical KS‐IRIS (triangle down) showed an up‐regulation of miR‐375, whereas a patient with unmasking KS (solid square) did not follow this trend (RQ < 1). These data indicate that circulating miR‐375 levels are down‐regulated in AIDS‐KS patients with complete clinical response, whereas they increase in patients not responding to cART, suggesting that circulating miR‐375 might be an indicator of active KS in AIDS patients

By comparing the miRNA profiles at baseline, that is naïve AIDS‐KS vs asymptomatic patients, 53 miRNAs were found to be differentially expressed (Table S2). Among the modulated miRNAs, two were up‐regulated in AIDS‐KS subjects and 51 down‐regulated (Figure 1A). cART response led to the modulation of 20 circulating miRNAs in asymptomatic patients; 15 were down‐regulated and 5 up‐regulated compared to baseline levels (Figure 1A and Table S3). Successful treatment in AIDS‐KS patients was associated with downmodulation of 6 miRNAs and up‐regulation of 11 miRNAs (Figure 1A and Table S4).

Among the differentially expressed miRNAs before and after cART, miR‐375 was one of the most markedly up‐regulated miRNAs in asymptomatic subjects (FC > 100) and the most markedly down‐regulated in AIDS‐KS patients (FC < –33) (Tables S3‐S4 and Figure 1A). Moreover, miR‐375 was the most up‐regulated (FC > 9) between naïve AIDS‐KS and naïve asymptomatic patients (Table S2 and Figure 1A). Therefore, miR‐375 was selected as a candidate miRNA with a potential prognostic value for further validation.

### 3.3 Validation

Patients selected for the validation showed a successful response to treatment, with a statistically significant decrease in HIV viraemia and a significant increment in CD4 cell counts (Figure S1). Pairwise comparison did not evidence statistically significant changes in all other parameters (not shown).

Validation was carried out by measuring miR‐375 levels in individual plasma samples. Among the three miRNAs found to be the most stable, miR‐92a was selected as reference miRNA to normalize miR‐375.

Comparison of the relative levels of miR‐375 among all four patient categories is shown in Figure 1B. Median levels of miR‐375 were higher in AIDS‐KS patients compared to asymptomatic subjects, suggesting that high levels of miR‐375 may be an indicator of active KS.

We then analysed validation data on individual patients with the same procedure used for the screening (fold change computation), and obtained the following results: naïve AIDS‐KS vs naïve asymptomatic patients, FC = 4.77; treated AIDS‐KS vs naïve AIDS‐KS, FC = –2.24; treated asymptomatic vs naïve asymptomatic, FC = 2.44. Albeit the levels of modulation were lower, trends observed in the screening were confirmed.

To better describe the modulation of miR‐375 during patient follow‐up, relative quantification (RQ) in each individual plasma of cART‐treated patients was calculated with respect to baseline, naïve condition (having an RQ = 1). Therefore RQ > 1 indicates miR‐375 up‐regulation, whereas RQ < 1 miR‐375 down‐regulation. As shown in Figure 1C, miR‐375 increased after cART in 9 of 11 asymptomatic patients (RQ > 1). Among the 11 AIDS‐KS patients showing complete clinical remission, seven demonstrated decreased levels of circulating miR‐375 (RQ < 1), whereas four had RQ > 1 (Figure 1C). To explain the discordant RQ values of the two asymptomatic patients and the four AIDS‐KS patients, we stratified all subjects according to RQ values > or <1, and analysed all the characteristics related to these patients. Interestingly, we found an association between RQ levels and CD4 cell counts. AIDS‐KS patients in trend with the screening, with RQ < 1, had significantly lower CD4 levels compared to AIDS‐KS patients not in trend, with RQ > 1, both at baseline (*P* = 0.0230) and after treatment (*P* = 0.0140) (Table 2, and data not shown). Conversely, asymptomatic patients showed the opposite (in trend with the screening, RQ > 1 and lower CD4 cell count; not in trend, RQ < 1 and higher CD4 cell count), although differences were not statistically significant (Table 2). Therefore, although limited to a small cohort, these findings might explain the distribution of RQ according to the CD4 levels.

**Table 2:** Analysis of CD4 cell count distribution according to RQ values of miR‐375

CD4 cell count (cells/µL)	Asymptomatic			AIDS‐KS		
	RQ < 1 (n = 2)	RQ > 1 (n = 9)	*P *value	RQ < 1 (n = 7)	RQ > 1 (n = 4)	*P* value
	Median (Q1;Q3)	Median (Q1;Q3)		Median (Q1;Q3)	Median (Q1;Q3)	
Naïve	657.5 (452;863)	345 (285;378)	0.0593	90 (50;230)	342.5 (241;571.5)	0.0230
cART‐treated	957 (606;1308)	671 (507;721)	0.3447	333 (160;390)	680 (511;745)	0.0140
Δ_(Treated‐Naïve)_	299.5 (154;445)	359 (80;410)	0.6374	160 (−20;264)	233.5 (100;343.5)	0.5074

CD4 cell counts are expressed as the median and interquartile range. RQ > 1 indicates miR‐375 up‐regulation and RQ < 1 indicates miR‐375 down‐regulation. The association between all parameters and RQ levels, dichotomized as higher and lower than 1, was verified with the Kruskal‐Wallis test. Naïve and cART‐treated AIDS KS patients showing down‐regulation of circulating miR‐375 (RQ < 1) had statistically significant lower amounts of CD4 lymphocytes compared to patients with RQ > 1. Naïve and cART‐treated asymptomatic patients with RQ > 1 had lower amounts of CD4 cells compared to patients with RQ < 1, although differences were not significant. These data, although limited to a few patients, suggest that modulation in the circulating amounts of miR‐375 may be associated with CD4 cell counts.

RQ, relative quantification; n, number of samples; Q1, first quartile; Q3, third quartile; cART, combined antiretroviral therapy; Δ_(Treated‐Naïve), _difference between cART‐treated and naïve condition.

In summary, validation carried out in individual plasma samples of HIV/HHV8‐infected patients in follow‐up confirmed the trends observed in the screening for most patients. Moreover, statistical analyses revealed an association between RQ levels and CD4 cell counts, thus explaining the discordant results. Therefore, these findings suggest that levels of miR‐375 might have a prognostic value in cART‐treated patients and might be associated with CD4 cell counts.

### 3.4 Analysis of miR‐375 in non‐responder AIDS‐KS patients

To further test the prognostic value of miR‐375 in AIDS‐KS patients, two patients with clinical PR and subsequent CR, and two patients with DP, who developed KS‐IRIS, were included in the follow‐up analysis (Table 1). As reported in Figure 1C, two patients showed increased levels of miR‐375 during PR (RQ > 1) (x and solid circle), and their RQs became <1 after CR. The patient who had paradoxical KS‐IRIS (triangle down) during cART showed an up‐regulation of miR‐375 (RQ > 1), whereas the patient with unmasking KS‐IRIS (solid square) did not follow this trend. As the unmasking condition is the development of KS during cART, the decrease in miR‐375 levels is the result of a transition between two rarely subsequent conditions, from naïve asymptomatic to cART‐treated AIDS‐KS. The comparison of cART‐treated AIDS‐KS vs naïve asymptomatic was carried out using low‐density array data (not shown) and gave the following negative fold changes with respect to the three normalizers: −2.56; −5.49; −3.17, suggesting that this rare manifestation of the disease might indeed be accompanied by a decrease in miRNA‐375.

Although limited to three non‐responder AIDS‐KS patients analysed in follow‐up during cART, these data might suggest that the persistence of high levels of miR‐375 in KS patients may be indicative of active disease.

## 4 DISCUSSION

Mir‐375 was previously shown to be involved in tumorigenesis, as it was found to be dysregulated in different tumour types, with levels of expression mainly down‐regulated in tumour tissues, suggesting that it may function as an oncosuppressor.^15^, ^16^ Conversely, circulating miR‐375 levels were found to be mainly increased in cancer patients,^17^, ^18^, ^19^ suggesting that miR‐375 may be indicative of oncogenic activity. In virus‐induced tumours, miR‐375 was shown to be up‐regulated in the peripheral compartment and in tumour tissues of Merkel cell carcinoma, indicating that circulating miR‐375 might be released by the tumour.^20^, ^21^ On the other hand, patients affected by hepatocellular carcinoma were found to have deregulated miR‐375 levels in neoplastic tissues and in serum samples, but the direction of dysregulation and virus association remain controversial.^19^, ^22^, ^23^

MiR‐375 was not reported to be among the miRNAs modulated in KS tissues.^6^, ^7^, ^8^ However, a common finding is a remarkable miRNA down‐regulation,^6^, ^7^, ^8^ involving also miRNAs acting as tumour suppressors, such as members of the let‐7 family. Our screening analysis showed that KS development in naïve patients is associated with downmodulation of many circulating miRNAs (Table S2 and Figure 1A), indicating a similar scenario in plasma and tumour. These data suggest that KS tumour and the other cell/s releasing miRNAs in the systemic compartment respond to similar signals, leading to miRNA shutdown. Consistently, two circulating miRNAs found in the screening, let‐7e and miR‐20a, were previously shown to be down‐regulated also in KS tissues.^6^, ^8^

The screening analysis on pooled plasma samples indicated miR‐375 as the most down‐regulated in AIDS‐KS patients and markedly up‐regulated in asymptomatic subjects after successful cART (Figure 1A). The validation carried out on individual plasma samples confirmed this opposite trend in most patients. MiR‐375 might be released by circulating cells and/or tissue reservoirs influenced by the tumorigenic activity and, possibly, by different signals in the absence of the neoplasia. In HIV/HHV8‐infected patients, regulation of miR‐375 levels might be affected by immunovirological parameters linked to HIV infection and by factors associated with HHV8, which differ between asymptomatic and AIDS‐KS patients. Statistical analyses showed that, after cART, CD4 cell counts in asymptomatic patients were significantly higher compared to AIDS‐KS patients in both screening and validation (Table S1). Despite the different therapy interval, this is in line with the lower CD4 cell recovery observed in HIV patients with more advanced immunodeficiency.^24^ Moreover, HHV8 antibodies titres were significantly higher in cART‐treated AIDS‐KS patients compared to asymptomatic subjects (Table S1), a finding consistent with the higher amount of lytic and latent antigens that the immune system encounters in patients with KS tumour compared to tumour‐free subjects. Indeed, HHV8 load, in PBMC and mainly in saliva, was higher in treated AIDS‐KS patients compared to asymptomatic patients, but the difference did not reach statistical significance (Table S1). Furthermore, analysis of the distribution of all patient characteristics according to the RQ levels demonstrated an association between RQ and CD4 levels (Table 2), that was statistically significant in naïve and treated AIDS‐KS patients. The association of miR‐375 changes and CD4 cell counts might explain the opposite trend between asymptomatic and AIDS‐KS patients and the discordant cases. Overall, our results suggest that circulating miR‐375 might have a prognostic value in cART‐treated patients, and that CD4 lymphocytes might be involved in miR‐375 regulation in HIV/HHV8‐infected subjects.

Validation results also indicated that circulating miR‐375 levels were higher in AIDS‐KS patients compared to asymptomatic patients, regardless of treatment (Figure 1B). Moreover, while tumour regression was associated with decreased miR‐375 levels (RQ < 1, Figure 1C) in AIDS‐KS patients with lower CD4 cell counts, at both baseline and after treatment (Table 2), tumour persistence during PR or DP in non‐responders was associated with up‐regulated miR‐375, suggesting that this miRNA could be a circulating marker of tumour activity. The patient that during DP showed decreased miRNA levels (RQ < 1) was a case of unmasking KS‐IRIS. The different trends shown by the two KS‐IRIS patients might be influenced by the different clinical presentation of this rare inflammatory condition, as paradoxical KS‐IRIS is characterized by the worsening of a pre‐existing tumour whereas unmasking KS‐IRIS leads to the development of KS, both in the context of a sudden immune reconstitution during cART.^25^ In the screening phase, the comparison of cART‐treated AIDS‐KS vs naïve asymptomatic patients showed a down‐regulation of miR‐375, consistently with the patient with unmasking KS‐IRIS, suggesting that this rare KS presentation might indeed lead to decreased miR‐375 amounts.

In conclusion, results of this pilot study suggest that circulating miR‐375 may have a prognostic value in HIV/HHV8‐infected patients, and might be influenced by CD4 cell counts. Moreover, miR‐375 might represent a good indicator for active KS in AIDS patients. Further studies in larger cohorts of patients are warranted to confirm these preliminary data.

## CONFLICT OF INTEREST

The authors declare that they have no competing financial interests.

## AUTHOR CONTRIBUTIONS

MAP, LG, and AM performed miRNA quantitative analyses. MAP and AM performed HHV8 serological and molecular analyses. AG performed the biostatistical analyses of microfluidic cards. PDB performed the statistical analyses of patients’ characteristics. MAP, LG, AG, PDB, PZ, AMC, LS, and MLC analysed and interpreted the data. AMC and LS enrolled and followed the study patients and analysed the clinical data. AG, PDB, LS, and PZ critically revised the manuscript. MAP, AMC, and MLC wrote the manuscript. MLC and AMC conceived the study. All authors read and approved the final manuscript.
