# Supplementary material for: Circulating miRNA‐375 as a potential novel biomarker for active Kaposi’s sarcoma in AIDS patients
Source: J Cell Mol Med. 2018 Dec 13;23(2):1486–94. doi: 10.1111/jcmm.14054 (PMC6349189; doi:10.1111/jcmm.14054)
Supplement: Supplementary file 1 [file JCMM-23-1486-s001.pdf]

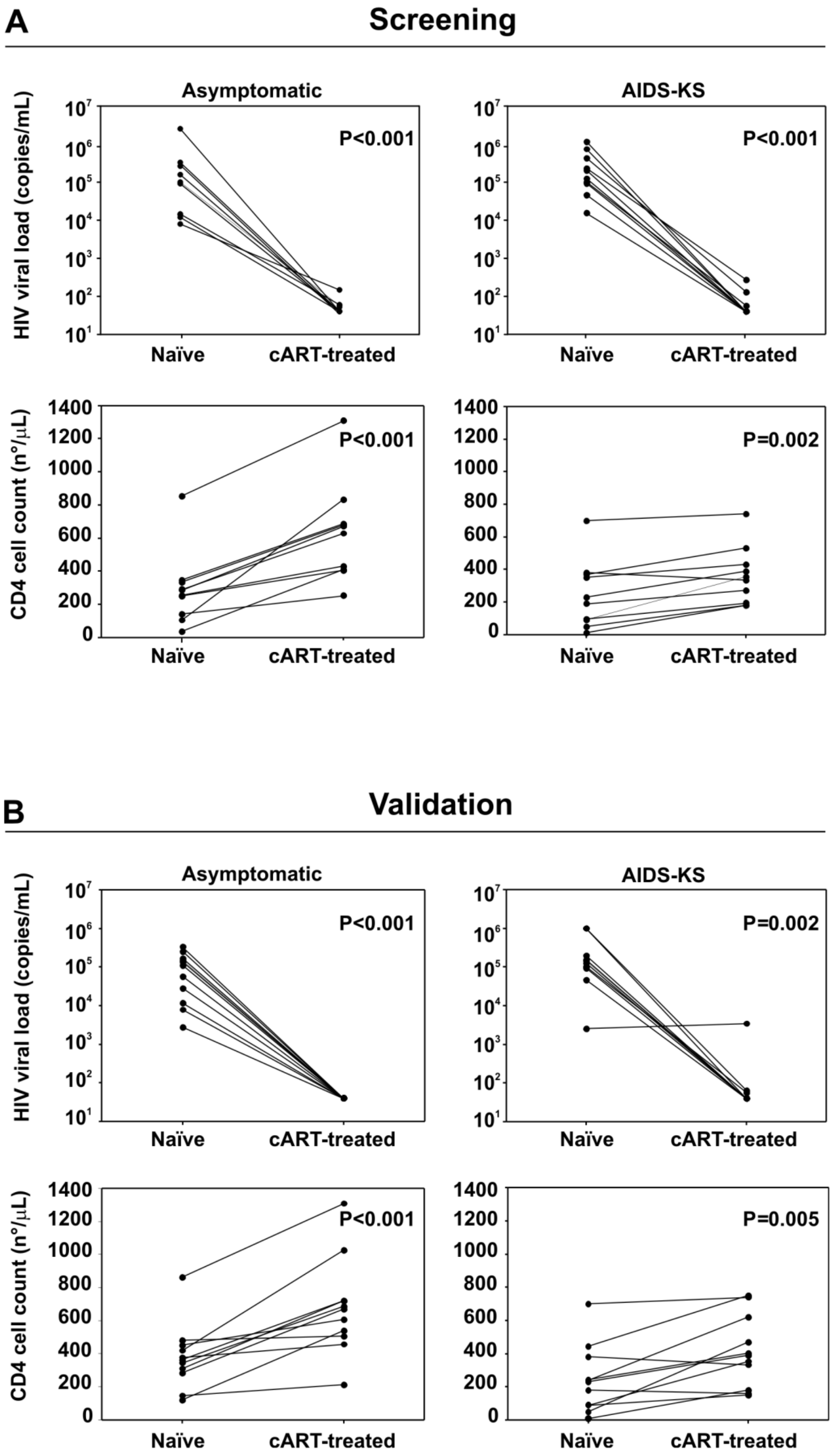
A
Screening
Asymptomatic
AIDS-KS
HIV viral load (copies/mL)
P<0.001
P<0.001
Naïve
cART-treated
CD4 cell count (n°/μL)
P<0.001
P=0.002
B
Validation
Asymptomatic
AIDS-KS
HIV viral load (copies/mL)
P<0.001
P=0.002
CD4 cell count (n°/μL)
P<0.001
P=0.005

Supplementary Figure 1. Trends of immunovirological parameters in the analyzed HIV/HHV8-infected patients. Changes in HIV viremia, expressed in copies/mL, and in CD4 cell counts, expressed in cells/µL, of individual samples of the two groups of patients analyzed in the screening (A) and validation (B) before and after cART. Following cART, all patients showed a statistically significant decrease in HIV viremia and a statistically significant increase in CD4 cell counts, and patients affected by AIDS-KS showed complete clinical response. The pairwise comparisons between levels at baseline and after therapy of HIV viremia and CD4 cell counts within each group of patients was based on the Wilcoxon signed rank test.
