# Supplementary material for: Circulating miRNA‐375 as a potential novel biomarker for active Kaposi’s sarcoma in AIDS patients
Source: J Cell Mol Med. 2018 Dec 13;23(2):1486–94. doi: 10.1111/jcmm.14054 (PMC6349189; doi:10.1111/jcmm.14054)
Supplement: Supplementary file 2 [file JCMM-23-1486-s002.pdf]

Supplementary Table 1| Comparison of all characteristics between asymptomatic and AIDS-KS patients of the screening and the validation

	**Naïve**			**Treated**			**$\Delta_{\text{(Treated-Naïve)}}$**		
	**Asymptomatic Median (Q1;Q3)**	**AIDS-KS Median (Q1;Q3)**	***p-value***	**Asymptomatic Median (Q1;Q3)**	**AIDS-KS Median (Q1;Q3)**	***p-value***	**Asymptomatic Median (Q1;Q3)**	**AIDS-KS Median (Q1;Q3)**	***p-value***
**Screening**									
**Age**	39 (34;43)	41.5 (30;53)	*0.5964*	40.5 (35;44)	42 (31;53)	*0.5963*			
**CD4 cell count (cells/µL)**	270 (141;332)	210 (90;370)	*0.7055*	650 (408;687)	343.5 (194;432)	*0.0233*	344.5 (174;386)	113.5 (80;160)	*0.0025*
**HIV plasma load (log_{10} copies/mL)**	4.86 (4.15;5.39)	5.2 (4.97;5.63)	*0.1988*	1.6 (1.6;1.72)	1.6 (1.6;1.76)	*0.8888*	-3.25 (-3.79;-2.43)	-3.43 (-3.71;-3.06)	*0.4497*
**HHV8 PBMC load (log_{10} GE/10^5 cells)**	0.72 (0.65;1.52)	1.32 (0.65;2.21)	*0.4476*	0.65 (0.65;0.65)	0.72 (0.65;1.26)	*0.0559*	-0.06 (-0.87;0)	0 (-1.34;0)	*1.0000*
**HHV8 saliva load (log_{10} GE/10^5 cells)**	1 (0.65;2.26)	1.62 (0.65;4.13)	*0.5456*	0.65 (0.65;3.45)	3.97 (2.94;4.83)	*0.0546*	0 (-0.69;0)	1.42 (0;2.3)	*0.0605*
**HHV8 ORF65 Antibodies**	45 (45;50)	400 (100;800)	*0.0012*	45 (45;100)	1200 (100;6400)	*0.0021*	0 (0;5)	600 (0;5600)	*0.0305*
**HHV8 LANA Antibodies**	150 (50;800)	1200 (400;3200)	*0.1102*	125 (50;1600)	3200 (1600;6400)	*0.0133*	0 (0;0)	0 (0;1500)	*0.2201*
**Treatment Months (Q1;Q3)**				20.5 (12;25)	10 (7;13)	*0.0628*			
**Validation**									
**Age**	36 (33;46)	38 (30;47)	*1.0000*	37 (35;47)	39 (31;48)	*0.9738*			
**CD4 cell count (cells/µL)**	362 (285;452)	230 (90;381)	*0.0709*	671 (507;721)	390 (181;620)	*0.0385*	359 (80;421)	160 (40;307)	*0.1076*
**HIV plasma load (log_{10} copies/mL)**	5.04 (4.08;5.23)	5.16 (4.97;6)	*0.2781*	1.6 (1.6;1.6)	1.6 (1.6;1.76)	*0.0695*	-3.44 (-3.62;-2.47)	-3.55 (-4.19;-3.24)	*0.2785*
**HHV8 PBMC load (log_{10} GE/10^5 cells)**	0.78 (0.65;1.45)	1 (0.65;1.23)	*0.8834*	0.65 (0.65;0.65)	0.95 (0.65;1.85)	*0.0822*	-0.12 (-0.79;0)	-0.19 (-1.37;0.62)	*0.9332*
**HHV8 saliva load (log_{10} GE/10^5 cells)**	1.96 (1.11;2.45)	1.62 (1.03;3.13)	*0.7933*	0.65 (0.65;2.75)	3.27 (2.62;4.3)	*0.1832*	-0.69 (-1.32;0)	0.66 (-0.42;1.69)	*0.1506*
**HHV8 ORF65 Antibodies**	45 (45;50)	400 (100;800)	*0.0006*	45 (45;50)	1600 (800;3200)	*0.0016*	0 (-5;0)	800 (400;2800)	*0.0076*
**HHV8 LANA Antibodies**	50 (50;1600)	2400 (400;12800)	*0.0206*	100 (50;1600)	1600 (1600;6400)	*0.0448*	0 (0;50)	0 (0;1200)	*0.8452*
**Treatment Months (Q1;Q3)**				19 (14;28)	9 (6;14)	*0.0103*			

Abbreviations: KS, Kaposi's sarcoma; Q1, first quartile; Q3, third quartile; HIV, Human immunodeficiency virus; HHV8, Human herpesvirus 8; PBMC, peripheral blood mononuclear cells; GE, genome equivalents; ORF65, open reading frame 65, encoding for a structural protein expressed during the lytic phase; LANA, latency-associated nuclear antigen; cART, combined antiretroviral therapy; $\Delta_{(\text{Treated-Naïve})}$, difference between cART-treated and naïve condition.

Medians and quartiles were calculated in all samples; data under detection level were replaced with a constant value (40 for HIV viremia, 4.5 Ge for HHV8 viral load, 45 for HHV8 antibody titers). Antibody titers were determined using 1:50 as first dilution, and are expressed as reciprocal of the highest dilution giving a positive result. In the validation phase, asymptomatic patients included 6 newly selected patients and 5 individuals of the screening analysis, whereas AIDS-KS subjects included 8 newly selected individuals. The differential distribution of all parameters at baseline, after therapy and their change from baseline, between AIDS-KS and asymptomatic patients was assessed by the Kruskal-Wallis test.
