# Supplementary material for: Circulating miRNA‐375 as a potential novel biomarker for active Kaposi’s sarcoma in AIDS patients
Source: J Cell Mol Med. 2018 Dec 13;23(2):1486–94. doi: 10.1111/jcmm.14054 (PMC6349189; doi:10.1111/jcmm.14054)
Supplement: Supplementary file 3 [file JCMM-23-1486-s003.pdf]

Supplementary Table 2 Differentially expressed miRNAs in naïve AIDS-KS *vs* naïve asymptomatic (group B *vs* group A) patients from TaqMan low-density array data.

miRNA ID	FC (hsa-miR-92a)	FC (hsa-miR-320)	FC (hsa-miR-484)
hsa-miR-598-4395179	-97.71	-63.23	-67.10
hsa-miR-195-4373105	-82.38	-53.31	-56.57
hsa-miR-155-4395459	-81.21	-52.55	-55.77
hsa-miR-133b-4395358	-48.06	-31.10	-33.00
hsa-miR-495-4381078	-46.41	-30.03	-31.87
hsa-miR-454-4395434	-32.56	-21.07	-22.36
hsa-miR-501-3p-4395546	-29.55	-19.12	-20.29
hsa-let-7e-4395517	-26.60	-17.21	-18.26
hsa-miR-26b-4395167	-22.61	-14.63	-15.53
hsa-miR-342-5p-4395258	-22.31	-14.44	-15.32
hsa-miR-95-4373011	-22.09	-14.29	-15.17
hsa-miR-29a-4395223	-21.98	-14.22	-15.09
hsa-miR-500-4395539	-21.43	-13.87	-14.72
hsa-miR-374a-4373028	-20.18	-13.06	-13.86
hsa-miR-381-4373020	-19.91	-12.88	-13.67
hsa-miR-29c-4395171	-19.81	-12.82	-13.61
hsa-miR-150-4373127	-17.87	-11.56	-12.27
hsa-miR-17-4395419	-13.49	-8.73	-9.26
hsa-miR-19a-4373099	-13.34	-8.64	-9.16
hsa-miR-374b-4381045	-13.14	-8.50	-9.02
hsa-miR-106a-4395280	-12.55	-8.12	-8.62
hsa-miR-590-5p-4395176	-12.30	-7.96	-8.44
hsa-miR-362-3p-4395228	-10.74	-6.95	-7.37
hsa-miR-342-3p-4395371	-10.48	-6.78	-7.20
hsa-miR-502-5p-4373227	-10.36	-6.71	-7.12
hsa-miR-483-5p-4395449	-8.96	-5.80	-6.15
hsa-miR-21-4373090	-8.95	-5.79	-6.15
hsa-miR-140-3p-4395345	-8.91	-5.77	-6.12
hsa-miR-28-3p-4395557	-8.68	-5.61	-5.96
hsa-miR-532-5p-4380928	-8.65	-5.60	-5.94
hsa-miR-340-4395369	-7.96	-5.15	-5.47
hsa-miR-20a-4373286	-7.87	-5.09	-5.40
hsa-miR-28-5p-4373067	-7.57	-4.90	-5.20
hsa-miR-191-4395410	-7.50	-4.86	-5.15
hsa-miR-19b-4373098	-7.33	-4.74	-5.03
hsa-miR-133a-4395357	-7.23	-4.68	-4.96
hsa-miR-27a-4373287	-6.57	-4.25	-4.51
hsa-miR-142-3p-4373136	-6.53	-4.23	-4.49
hsa-miR-106b-4373155	-6.48	-4.20	-4.45
hsa-miR-15b-4373122	-6.40	-4.14	-4.39
hsa-miR-100-4373160	-6.35	-4.11	-4.36
hsa-miR-186-4395396	-6.29	-4.07	-4.32

hsa-miR-26a-4395166	-6.27	-4.06	-4.30
hsa-miR-660-4380925	-6.06	-3.92	-4.16
hsa-miR-451-4373360	-5.92	-3.83	-4.07
hsa-miR-324-3p-4395272	-5.80	-3.75	-3.98
hsa-miR-140-5p-4373374	-5.73	-3.71	-3.93
hsa-miR-339-3p-4395295	-5.46	-3.53	-3.75
hsa-miR-93-4373302	-5.41	-3.50	-3.71
hsa-miR-16-4373121	-5.10	-3.30	-3.50
hsa-miR-222-4395387	-5.09	-3.30	-3.50
hsa-miR-485-3p-4378095	5.53	8.54	8.05
hsa-miR-375-4373027	9.00	13.90	13.10

FC: fold change. FCs are calculated with respect to the three most stable miRNAs, reported between parentheses. Downregulated miRNAs are highlighted in light blue, whereas upregulated ones in light red.

Supplementary Table 3 Differentially expressed miRNAs in cART-treated *vs* naïve asymptomatic patients (group A), from TaqMan low-density array data.

miRNA ID	FC (hsa-miR-92a)	FC (hsa-miR-320)	FC (hsa-miR-484)
hsa-miR-10a-4373153	-61.77	-36.20	-48.62
hsa-miR-95-4373011	-51.74	-30.32	-40.72
hsa-miR-500-4395539	-33.81	-19.81	-26.61
hsa-miR-195-4373105	-28.01	-16.42	-22.05
hsa-miR-342-5p-4395258	-24.05	-14.09	-18.93
hsa-miR-483-5p-4395449	-20.11	-11.79	-15.83
hsa-miR-502-5p-4373227	-16.35	-9.58	-12.87
hsa-let-7e-4395517	-13.67	-8.01	-10.76
hsa-miR-150-4373127	-8.74	-5.12	-6.88
hsa-miR-21-4373090	-8.32	-4.88	-6.55
hsa-miR-148a-4373130	-7.66	-4.49	-6.03
hsa-miR-142-5p-4395359	-7.40	-4.33	-5.82
hsa-miR-193a-3p-4395361	-6.68	-3.92	-5.26
hsa-miR-132-4373143	-6.21	-3.64	-4.88
hsa-miR-532-3p-4395466	-5.06	-2.96	-3.98
hsa-miR-221-4373077	7.32	12.50	9.31
hsa-miR-370-4395386	32.83	56.02	41.71
hsa-miR-210-4373089	48.12	82.11	61.14
hsa-miR-375-4373027	106.71	182.09	135.58
hsa-miR-22-4373079	181.46	309.64	230.56

FC: fold change. FCs are calculated with respect to the three most stable miRNAs, reported between parentheses. Downregulated miRNAs are highlighted in light blue, whereas upregulated ones in light red.

Supplementary Table 4 Differentially expressed miRNAs in cART-treated *vs* naïve AIDS-KS patients (group B) from TaqMan low-density array data.

miRNA ID	FC (hsa-miR-92a)	FC (hsa-miR-320)	FC (hsa-miR-484)
hsa-miR-375-4373027	-44.50	-37.86	-33.93
hsa-miR-202-4395474	-21.04	-17.91	-16.05
hsa-miR-382-4373019	-9.72	-8.27	-7.41
hsa-miR-885-5p-4395407	-8.30	-7.06	-6.33
hsa-miR-296-5p-4373066	-7.49	-6.38	-5.71
hsa-miR-381-4373020	-6.31	-5.37	-4.81
hsa-miR-29c-4395171	5.08	5.97	6.67
hsa-miR-374a-4373028	5.30	6.23	6.95
hsa-miR-28-5p-4373067	6.65	7.81	8.72
hsa-miR-203-4373095	6.88	8.08	9.02
hsa-miR-330-3p-4373047	6.98	8.20	9.15
hsa-miR-455-3p-4395355	7.60	8.93	9.97
hsa-miR-495-4381078	7.80	9.16	10.23
hsa-miR-598-4395179	13.06	15.35	17.13
hsa-miR-454-4395434	15.36	18.05	20.14
hsa-miR-886-3p-4395305	36.57	42.98	47.97
hsa-miR-133b-4395358	39.31	46.19	51.55

FC: fold change. FCs are calculated with respect to the three most stable miRNAs, reported between parentheses. Downregulated miRNAs are highlighted in light blue, whereas upregulated ones in light red.
